# Whole-genome bisulfite sequencing of goat skins identifies signatures associated with hair cycling

**DOI:** 10.1186/s12864-018-5002-5

**Published:** 2018-08-28

**Authors:** Chao Li, Yan Li, Guangxian Zhou, Ye Gao, Sen Ma, Yulin Chen, Jiuzhou Song, Xiaolong Wang

**Affiliations:** 1 College of Animal Science and Technology, Northwest A&F University, Yangling, 712100 China; 2 Department of Animal and Avian Sciences, University of Maryland, College Park, MD 20742 USA

**Keywords:** DNA methylation, Hair follicle, WGBS, Hair growth, Epigenetic regulation, Goat

## Abstract

**Background:**

Hair follicles (HFs), upon development, undergo repetitive cycles of growth (anagen), regression (catagen), and rest (telogen). The transition between the stages is determined by multiple molecular signals, including DNA methylation, which plays important roles in mammalian cellular identity and is essential for the development of HFs. Secondary hair follicles (SHFs) in cashmere goat exhibit classic cyclic hair development, and little has been done on a genome-wide scale to examine potentially methylated genes involved in the hair cyclic transition.

**Results:**

Genome-wide DNA methylation profiles between skin tissues sampled during the anagen and telogen stages in cashmere goats were investigated using whole-genome bisulfite sequencing (WGBS). The methylation status was observed to be higher in the skin samples with HFs in the telogen than those in the anagen stage. A total of 1311 differentially methylated regions (DMRs) were identified between the two groups, which contained 493 fully annotated DMR-related genes (DMGs) (269 Hyper- DMGs and 224 Hypo-DMGs). Furthermore, a significant over-representation of the functional categories for DMGs related to immune response and intercellular crosstalk during hair cycling was observed. By integrating DNA methylation and mRNA expression data, we revealed that four genes (*FMN1*, *PCOLCE*, *SPTLC3*, and *COL5A1*) are crucial factors for elucidating epigenetic mechanisms contributing to the telogen-to-anagen transition.

**Conclusion:**

Our study provided systematic methylome maps pertaining to the hair cycling stages (anagen vs telogen) at a single-base resolution, and revealed stage-specific methylation loci during cashmere growth or quiescence. Furthermore, we identified epigenetically regulated genes that are potentially involved in HF development and growth in cashmere goats, and likely in other mammal species.

**Electronic supplementary material:**

The online version of this article (10.1186/s12864-018-5002-5) contains supplementary material, which is available to authorized users.

## Background

Epigenetic mechanisms play crucial roles in the regulation of a variety of biological processes involved in skin development, regeneration, aging [1–4], and cycling of hair follicles (HFs) [5, 6]. Of all the epigenetic processes, DNA methylation is a key epigenetic factor in the regulation of gene expression, which consequently alters cellular phenotypes. The ablation of DNA methyltransferase 1 in mouse skin resulted in changes to the epidermal thickness and significant reduction in HF size, number and cycling activity. These observations suggest an essential role of DNA methylation in the regulation of stem cell homeostasis during the development and maintenance of ectodermal organs [7]. The deletion of *Hdac1*, a histone deacetylase, resulted in the upregulation of *p16/INK4a,* which could stimulate hair matrix keratinocyte proliferation, resulting in abnormal hair growth [8]. By examining the role of chromatin modifications in adult stem cells from well-characterized HFs, Lien et al. (2011) demonstrated the ability of HF stem cells to induce self-renewal without differentiation [9]. Moreover, aging and environmental factors (e.g. sun exposure) are significant factors in the phenotypic changes associated with skin aging in humans [10]. Large blocks of hypomethylated genes are found in the older, greater sun-exposed epidermis, and the degree of hypomethylation has been observed to be highly correlated with clinical measures of photo-aging [11].

Hair growth is the product of synthetic processes carried out by HFs, which are embedded in the skin, and is a unique characteristic of mammals. Hair fiber is an important commercial product harvested from fiber producing animals, such as sheep and goats. The hair renewal cycle in cashmere goats is an annual occurrence. Briefly, the primary HFs develop in the skin and produces guard hair. The secondary HFs produce the fine fibers, known as cashmere, in a seasonal and cyclical manner over the lifetime of the animal. The stages of this cyclic process are referred to as anagen (growth), catagen (regression), and telogen (rest), during which several morphological changes take place [12, 13]. The regular periodic changes in cashmere goat skin makes it an attractive animal model to investigate cyclic changes of genetic regulation. Previously, we identified a number of genetic determinants (mainly genes and miRNAs) that potentially control HF development and growth through analysis of skin tissues from the 3 different HF cycling stages in cashmere goats [14–17]. However, a solid understanding of the epigenetic factors, especially DNA methylation, functioning in the regulation of HF biology in cashmere goats remains elusive.

In the present study, whole genome bisulfite sequencing (WGBS) was conducted to dissect out the patterns of epigenetic regulation associated with the differentiation of HFs in cashmere goats. Several differentially methylated regions (DMRs) were identified, as were genes that appear to be important factors in the regulation of hair cycles in the anagen and telogen stages. The data identified specific epigenetically regulated pathways associated with HF development, and suggested that epigenetic changes could underpin the conversion of hair cycling in fiber-producing animals.

## Results

### Genome-wide DNA methylation profiling in goat skins

**Fig. 1:**
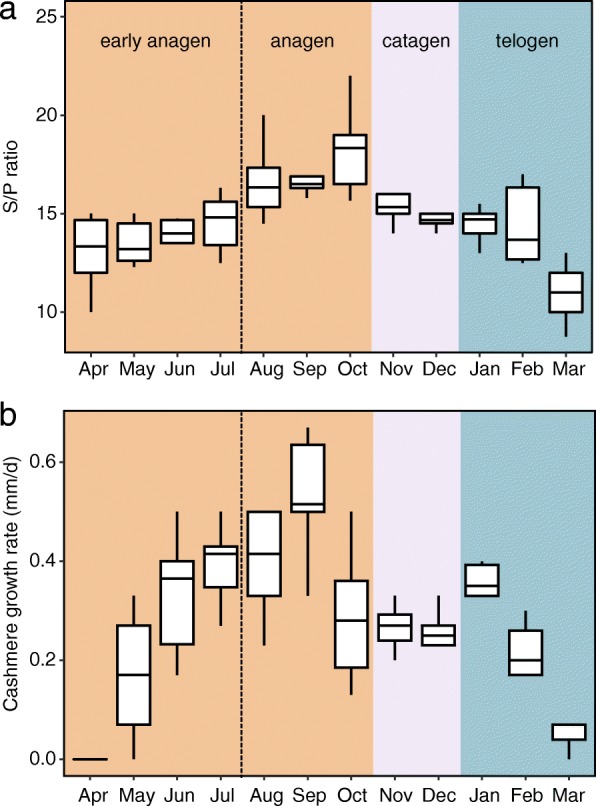
The skin secondary to primary follicles (S/P) ratio (**a**) and cashmere growth rate (**b**) in cashmere goats annually

To determine the hair cycling stages in the skins from the cashmere goats used in the present study, skin tissues were sampled at key points over the course of a year for H&E staining (Additional file 1: Figure S1). The ratio of secondary to primary follicles (S/P) was calculated, in addition to cashmere growth rate (Fig. 1). These data indicated that the skin samples collected in October were representative of the anagen stage, whereas samples collected in March were in the telogen stage. Therefore, paired tissue samples of the same animals (*n* = 3) were collected in October and March for further analysis by WGBS.

A total of 265.24 G and 252.77 G raw data were generated for the anagen and telogen groups, respectively. An average of 295, 280 million raw reads of WGBS data for the anagen and telogen groups (Table 1) were analyzed. Approximately 77.9% (anagen) and 80.9% (telogen) of clean reads could be independently mapped to the goat reference genome assembly CHR2.0 [18] (Table 1). Any ambiguously mapped and duplicate reads were removed from further analysis.

**Table 1:** Summary of WGBS dataset

Sample	Raw reads	Clean reads	Mapped reads	Mapping rate (%)	Duplication rate (%)	Raw data (G)	Clean data (G)	Clean ratio (%)
anagen_1	310,947,773	298,040,197	229,252,519	76.92	11.14	93.28	82.80	88.77
anagen_2	305,862,462	290,303,912	224,056,559	77.18	11.05	91.76	80.86	88.12
anagen_3	267,339,138	254,618,756	202,676,529	79.60	10.04	80.20	70.66	88.10
telogen_1	271,040,541	258,315,532	207,349,877	80.27	10.83	81.31	71.96	88.50
telogen_2	305,284,706	290,618,748	230,547,852	79.33	10.56	91.59	81.10	88.55
telogen_3	266,241,455	263,463,096	218,674,369	83.00	11.36	79.87	76.71	96.04

### DNA methylation patterns in the goat genome

**Fig. 2:**
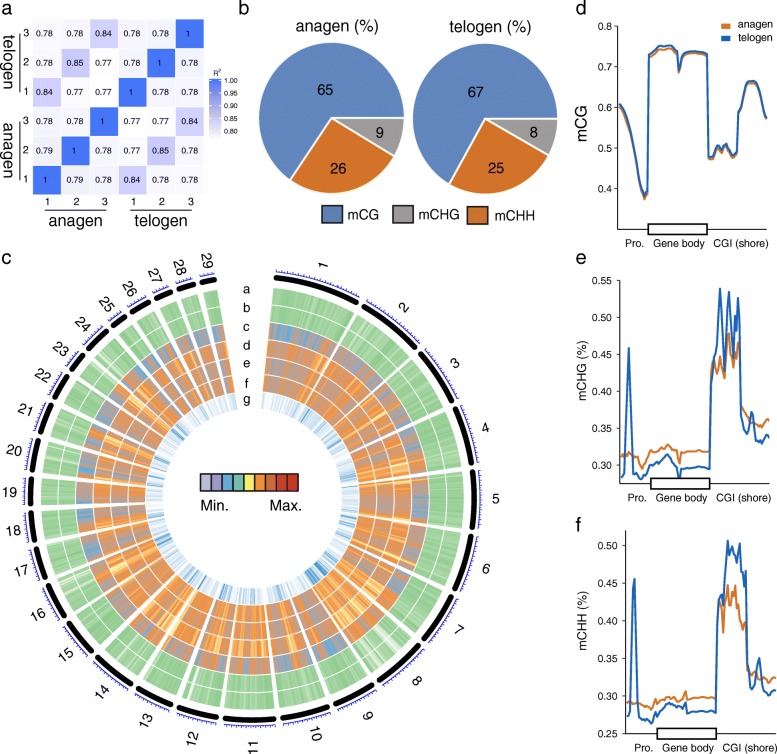
Methylation profiles of skins samples in anagen and telogen stages in cashmere goats. **a** Pearson correlation of base resolution of CpG methylation genome-wide between the goat skin anagen and telogen samples. **b** The proportion of of mCs (mCG, mCHH and mCHG) in anagen and telogen tissue samples. **c** Circos plot representing the methylation density of cashmere goat chromosomes. Track order: (a) mCG_anagen; (b) mCG_telogen; (c) mCHH_anagen; (d) mCHH_telogen; (e) mCHG_anagen; (f) mCHG_telogen; (g) gene density. Heatmap representation of the average methylation densities in 300 kb windows independent of sequence context. The CG (**d**), CHG (**e**), and CHH (**f**) methylation levels between the anagen and telogen groups at different sequence regions. Pro. indicates protomer

To assess the similarity of the three skin samples within each group, pairwise Pearson correlation coefficients were calculated for every two samples. The correlation coefficients were observed to be > 0.77 in the anagen samples and > 0.84 in the telogen samples (Fig. 2a), indicating a higher consistency of samples used for WGBS analysis. Subsequently, the methylation levels of each cytosine were calculated. An average of 50,182,060 and 48,109,807 methylated cytosines (mCs) in the anagen and telogen stages, respectively were detected (Additional file 2: Table S1). In each group, ~ 4.2–4.3% of all genomic C sites were methylated (Additional file 2: Table S2). Methylation in goats was found to exist in three classifications: mCG, mCHH (where H is A, C, or T), and mCHG. The classifications of mCs indicated a similar proportion between the anagen and telogen groups (Fig. 2b). To investigate the changes methylation on a genome-wide scale, methylation along all chromosomes were assessed using a 300-kb sliding window (Fig. 2c). The observed differences in CG methylation were negligible. Next, correlations between methylation levels and genomic features were analyzed. Methylation levels were positively correlated with chromosomal length (Pearson’s *r* = 0.69, *P* < 10^− 4^) and repeat number (*r* > 0.55, *P* < 0.01), but were negatively correlated with the CpG contents (*r* > 0.94, *P* < 10^− 4^), ratios between the observed and expected numbers of CpG sites (CpG_o/e_) (*r* > 0.96, *P* < 10^− 16^), as well as the number of genes (*r* > 0.80, *P* < 10^− 6^) for both the anagen and telogen groups (Additional file 1: Figure S2).

To examine the overall methylation status, levels of methylation in 5 genetic structural regions were determined, including promoters, exons, introns, CpG islands (CGIs) and CGI shores (regions within 2 kb of an island). At the genome-wide scale, similar CG methylation levels were observed between the samples from anagen and telogen stages. However, the telogen samples exhibited a slightly higher methylation status in all regions (Fig. 2d). Interestingly, a marked hypomethylation was observed in the regions surrounding transcription start site. Similar methylation patterns have been reported in other animal species, such as cattle [19], sheep [20] and pigs [21]. The CHG and CHH methylation levels demonstrated similar trends. Notably, the levels of methylation in telogen samples peaked in the promoter region, then dropped after the transcription start sites, and later increased again after the transcription termination sites (Fig. 2e, f). However, the CHG and CHH methylation levels in telogen samples at gene bodies were lower than was observed in anagen samples. Remarkably, a distinct increase in CHG and CHH methylation levels at CGI and CGI shores in both groups was observed, while the methylation level in the telogen group was clearly higher than in the anagen group (Fig. 2e, f).

### Identification of DMRs and functional enrichment of methylated genes

**Fig. 3:**
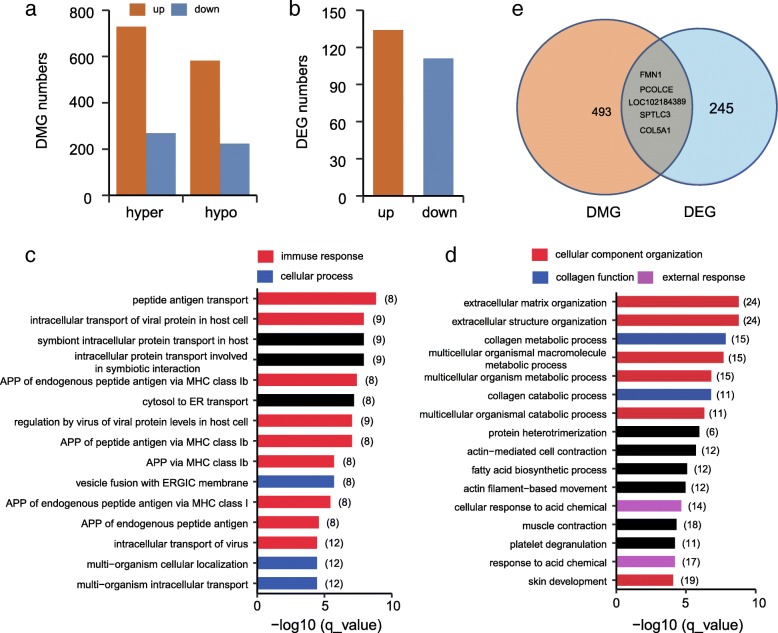
**a** The number of differentially methylated genes (DMGs) as determined by WGBS. **b** The number of differentially expressed genes (DEGs) as determined by RNA-seq. Top Gene Ontology (GO) terms enriched using DMGs **c** and DEGs **d**. **e** Overlap of DMGs and DEGs

To identify genomic regions with different levels of methylation between the anagen and telogen stages, methylated residues were examined by analyzing sliding windows of 1000 bp in length using the BSmooth approach [22]. A total of 1311 differentially methylated regions (DMRs) were identified, including 729 hyper DMRs (in telogen samples) and 582 hypo DMRs (Additional file 2: Table S3). Next, the genes within the DMRs were annotated using the ARS1 assembly [23]. The analysis revealed a total of 493 genes that were determined to be differentially methylated genes (DMGs), encompassing 269 hyper DMGs and 224 hypo DMGs (Fig. 3a, Additional file 2: Table S3).

To obtain a better mechanistic understanding of the gene networks that may be responsible for functional differences during the transition of hair growth phases, Gene Ontology Biological Process (GO-BP) enrichment analysis was performed to reveal over-represented categories for annotated DMGs using g:Profiler [24]. The DMGs were found to be significantly over-represented for functional categories including immune response (e.g. peptide antigen transport, antigen processing and presentation) and cell communication (e.g. multi-organism cellular/intracellular transport and localization) (Fig. 3c; Additional file 2: Table S4). These results highlight the central roles of intercellular crosstalk and signaling transduction in epigenetic regulation of hair cycling.

### Integrated analysis of WGBS and RNA-seq data

To better understand the functions of the genes involved in telogen-to-anagen transition in goat HFs, we reanalyzed the skin transcriptome data we have generated previously [14]. A total of 245 differentially expressed genes (DEGs) between the telogen and anagen stages were observed using EdgeR [25] with the new genome assembly ARS1 [23]. These DEGs included 134 genes that were up-regulated, and 111 that were down-regulated (Fig. 3b, Additional file 2: Table S5). The top 10 DEGs consisted of three collagen genes (*COL3A1*, *COL1A2*, and *COL1A1*), and the desmogleins family member gene DSG1. The most significant DEG was the *TCHH* gene, which has been associated with a straight hair phenotype in humans [25]. Interestingly, this gene was also identified in hair fibers using iTRAQ approach from another recently published study [26]. GO enrichment analysis indicated that these DEGs could be involved in multicellular organismal processes, skin development, fatty acid metabolism (e.g. fatty acid biosynthetic/metabolic process, regulation of lipid biosynthetic/metabolic process), and response to stimulus (e.g. response to acid chemical/peptide/metal ion/extracellular stimulus) (Fig. 3d, Additional file 2: Table S6). The functional enrichment results are in agreement with results of previous RNA-seq studies using skin tissues from cashmere goats [13, 14].

**Fig. 4:**
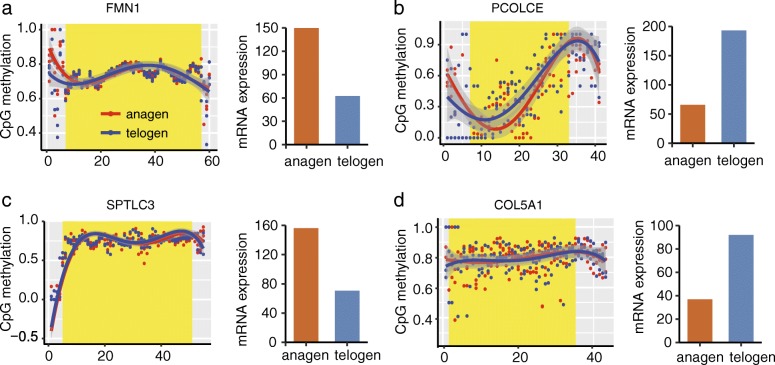
Correlation of DNA methylation and gene expression between the anagen and telogen stages in four genes. **a** *FMN1*; **b** *PCOLCE*; **c** *SPTLC3*; **d** *COL5A1*. Yellow shadow represents the gene coding areas

To determine the relationship between DNA methylation and gene expression, 5 overlapping genes (*FMN1*, *PCOLCE*, *SPTLC3*, *COL5A1*, and *LOC102184389*) between the 493 DMGs and 245 DEGs were analyzed (Fig. 3e). The methylation data were extracted and examined the overall DNA methylation status in the four annotated DMGs (*FMN1*, *PCOLCE*, *SPTLC3*, and *COL5A1*). However, no correlation was detected between the methylation and expression status. Specifically, significant methylation differences were found in their introns of two hypermethylated genes (*FMN1* and *PCOLCE*), but the mRNA expression was variable (Fig. 4). A similar pattern was observed in two hypomethylated genes (*SPTLC3* and *COL5A1*). These results are inconsistent with the notion that the methylation of promoter or gene body DNA is correlated with gene expression [27, 28]. However, our results are consistent with the findings reported in other recent WGBS studies [29, 42], in which the regulation of methylation and expression is diverse. This is likely due to the fact that the methylation differences observed were predominantly found in the introns of CGI in the present study (Additional file 2: Table S3). Therefore, the intragenic methylation of DNA might be involved the regulation of alternative splicing [30].

## Discussion

As an optimally tractable model system, the HF consists of multiple different cell populations, including those of neural crest, ectodermal or mesodermal origin [31]. The HF is a regenerating system that undergoes continuous cycle consisting of a growth phase (anagen), a relative ‘resting’ phase (telogen), and organ regression (catagen) stage. The study of hair cycles thus provides a model system through which the dynamic nature of the HF with drastic methylation changes associated with the stage of hair cycle can be studied. In the present study, for the first time to our knowledge, the hair cycles were studied using genome-level profiles of DNA methylation with single-base resolution in animal skins. As a result, a set of genes that were identified that contained significant changes to the DNA methylation statuses. These genes are potentially important for the elucidation of epigenetic mechanisms that underlie the development and maintenance of HFs.

Overall, the gene bodies (exons and introns) exhibited the highest levels of CG methylation, relative to the gene promoters in skin tissues during the anagen or telogen stages (Fig. 2c). These observations are consistent with previous studies on gene methylation status in other livestock species, including goats [32], horses [33] and cattle [19]. Similar patterns were observed in a very recent goat study [32], in which the distribution of methylated residues was higher in gene bodies than upstream regions in caprine hypothalamus and ovary tissues. In the present study, CpG islands and CGI shores showed a higher CGI and CGI methylation densities than gene bodies and promoter regions, respectively. These data are also consistent with the findings in a study of caprine hypothalamus and ovary tissues, which reported that approximately 50% of the CGIs in are methylated [32]. Although there were no apparent CG methylation differences between the groups, the overall methylation level (including mCHG and mCHH) of the telogen group was higher than that of the anagen group. A miniorgan, HF, undergoes repetitive cycles through the transition from the resting telogen to the active anagen stage. The HFs are largely quiescent during the telogen stage by exhibiting shortened and condensed HFs in the dermis [34]. This could explain the global reduction in transcription of genes during the telogen stage, which could be a result of a relatively higher proportion of methylated residues. Similarly, an increase in methylation status was reported in quiescent telogen bulge stem cells than was observed in activated anagen bulge stem cells derived from skin cells in mice [35].

Of the 1311 significant DMRs, 493 DMR-related genes were annotated through further analysis. The GO functional analysis performed in the present study demonstrated that these DMGs were primarily enriched in the categories of immune response (e.g. peptide antigen transport, antigen processing and presentation) and cell communication (e.g. multi-organism cellular/intracellular transport and localization) (Fig. 3d). These results are consistent with previous reports of GO enrichment using DEGs obtained from different types of skin cells [36], or DEGs during specific phases of hair growth [37]. Furthermore, the data presented here in addition to the referenced studies highlights the important roles of DNA methylation in hair cycling, especially in the transition of HF developmental stages.

The methylation of DMGs was correlated with gene expression levels, most likely through the mechanism of transcriptional repression. In the present study, potential functionally relevant DNA methylation changes were identified through an analysis of the correlation between DNA methylation profiles and RNA-seq data. This analysis identified five overlapping genes (*FMN1*, *PCOLCE*, *SPTLC3*, *LOC102184389*, and *COL5A1*). Of these four annotated genes, the *FMN1* gene interacts with α-catenin, and is essential for the formation and maintenance of adherens junctions in keratinocytes [38]. the *PCOLCE* gene is highly expressed in the dermal papilla cells derived from skin [39], and functions as an activator of BMP1 in the BMP signaling pathway [40]. GREM1, an inhibitor of BMP, has been implicated as a key regulator during HF development [9, 41]. The *SPTLC3* gene encodes lipid-metabolizing enzymes in the ceramide biosynthesis pathway [42], and has been associated with lipid metabolism in epidermal tissue. The *COL5A1* gene functions to orchestrate reorganization within the skin structure, and knockouts of *COL5A1* resulted in an increased skin stretching phenotype in mice [43]. These results suggest that these 4 genes are strong potential targets through which the epigenetic mechanisms guiding the transitions of HF between growth and quiescence can be elucidated in cashmere goats.

## Conclusions

Through the WGBS approach, stage-specific DNA methylomes were characterized during a life cycle of skin HFs in a cashmere goat model at a single-base resolution. We identified several DMRs between the anagen and telogen stages in HFs, and revealed increased methylation in the telogen stage. In addition, it was observed that a total of 493 genes were methylated, and these genes are primarily involved in the processes of immunity and cellular communication. The data presented here suggests a role for methylation dynamics in the regulation of hair cycling in mammals, specifically cashmere goats, which could potentially serve as a model organism for hair renewal studies in humans.

## Methods

### Animals and sample preparation

Skin tissues were obtained from three female adult goats (2 year-old) as previously described [15]. The HF developmental stages were determined by H&E staining, S/P ratio, and the growth rate of cashmere fibres. Remaining skin tissue samples were rinsed in ice-cold DEPC-treated water and were cut into small pieces, and then preserved in RNALater (ABI, USA) for storage at − 70 °C until further processing. Genomic DNA was extracted from skin samples using the Qiagen DNeasy Blood & Tissue Kit (Qiagen) according to the manufacturer’s instructions. The resulting DNA was treated with RNase to degrade any remaining cellular RNA. The integrity and quality of DNA were evaluated using an Agilent 2100 bioanalyzer (Agilent Technologies, Palo Alto, CA) prior to analysis. The entire experimental procedure was approved and supervised by the Animal Care Commission of Northwest A&F University under contract (2011–31,101,684).

### WGBS library preparation, sequencing, quality analysis and mapping

Three replicated samples from the anagen and telogen stages were used for library preparation. WGBS DNA libraries were prepared following a previously described method [29]. Briefly, DNA (5 μg) spiked with 26 ng of lambda DNA was fragmented by sonication to produce fragments of 200–300 bp with the Covaris S220 Focused-ultra sonicator (Covairs, Woburn, MA). Next, end repair and adenylation reaction were performed. Cytosine-methylated barcodes were then ligated to the DNA fragments. These DNA fragments were treated twice with bisulfite using the EZ DNA Methylation-GoldTM Kit (Zymo Research, Orange, CA). The resulting single-strand DNA fragments were amplified by PCR using the KAPA HiFi HotStart Uracil + ReadyMix (2×). A Qubit® 2.0 Fluorometern was utilized to quantify the concentrations of DNA in the library. The insert size was assessed on an Agilent Bioanalyzer 2100 system (Agilent Technologies, Palo Alto, CA). After clustering of the index-coded samples was completed using a cBot Cluster Generation System via TruSeq PE Cluster Kit v3-cBot-HS, the libraries were sequenced on an Illumina HiSeq 2500 platform (Novogene Bioinformatics Institute, Beijing, China).

Subsequently, 100 bp/50 bp single-end reads were generated. Image analysis and base calling was performed using the standard Illumina pipeline. Finally, 100 bp paired-end reads were generated. Clean reads were obtained using the following 3 steps: (1) each read was scanned for the 3′ adapter oligonucleotide sequence. If detected, the read was removed. (2) The percentage of Ns (unknown bases) in each read was calculated. If the percentage was < 10%, the read was removed. (3) Low quality reads (PHRED score ≤ 5, percentage of low quality bases ≥50%) were trimmed. In addition, the Q20, Q30, and GC contents of the clean reads were calculated. All downstream analyses were based on the good-quality clean reads. The goat reference genome [23](https://www.ncbi.nlm.nih.gov/genome/?term=goat) was transformed into the bisulfite-converted version (C-to-T and G-to-A converted), and was indexed by Bowtie 2. Clean reads were also transformed into the fully bisulfite-converted version, and then aligned to the above converted version of the genome. Reads that aligned to the same regions of the genome were regarded as duplicates. The sequencing depth and coverage are summarized with duplicate reads removed.

### Identification of differentially methylated regions

The “bsseq” package (http://www.bioconductor.org/packages/release/bioc/html/bsseq.html) under the R environment was used to identify DMRs across the full genome using the sliding window approach, the BSmooth algorithm [22]. Briefly, the sliding window size and step were set at 1000 and 100 bp, respectively. Genomic regions with different levels of methylation or fold changes being greater than the cut-off values, and the number of cytosines contained within the region being greater than the cut-off value considered to be DMRs. The resulting DMRs were verified by statistical means, using a student’s t-test. A FDR value of < 0.05 was used to determine specific cut-off values. The methylated cytosine loci were merged as DMR according to 1) the average methylation levels between 2 pairwise groups was > 0.25; 2) a single DMR should contain a minimum of 3 differentially methylated sites; 3) the distance between two cytosines is > 300 bp; and 4) the potential DMRs within one region were merged as a single DMR.

### Functional enrichment analysis

Functional enrichment analysis of GO was performed using the using the g:Profiler [24], for the methylation related genes. The GO terms were corrected using the Benjamini-Hochberg method, and such genes with a corrected *P*-value of less than 0.05 were considered to be significantly enriched.

### Gene expression data re-analysis

Previously, we performed RNA-seq analysis of goat skin samples representing the three stages of hair cycles (anagen, catagen, and telogen). The sequences were then aligned to the *Bos taurus* genome sequence assembly [14], as the *Capra hircus* reference sequence was not available at the time. Among the raw data from RNA-seq, the sequencing adaptors, reads with greater than 5% of nucleotides being unknown, and low quality reads (more than half of the base quality scores were less than 10) were removed. The remaining clean data were mapped to the currently available goat genome sequence assembly (ARS1) [23] in order to screen for DEGs. Read counts for each gene were calculated using the HTSeq package for Python [44]. Determination of DEGs between the anagen and telogen samples was determined using the EdgeR Bioconductor package [25]. DEGs were defined as Q value≤0.05 and absolute value of|log (fold change)| > 1.

## Additional files

Additional file 1: **Figure S1.** H&E staining of goat skins at different stage. **Figure S2.** The correlation between methylation levels and chromosome length, CpG content, the ratio between the observed and expected numbers of CpG sites (CpG_o/e_), gene number and repeat number. (DOCX 1186 kb)

Additional file 2: **Table S1.** Methylated cytosines in the skins at anagen and telogen stages. **Table S2.** The proportion of methylated cytosines in goat skins at anagen and telogen stages. **Table S3.** The identified DMRs through WGBS and the annotated genes. **Table S4.** GO biological process terms enriched for annotated DMGs. **Table S5.** DEGs identified in the cashmere goat skins at anagen and telogen stages. **Table S6.** GO biological process terms enriched for DEGs revealled in goat skins. (XLS 256 kb)

## Abbreviations

CGIs: CpG islands
DEGs: Differentially expressed genes
DMGs: DMR-related genes
DMRs: Differentially methylated regions
GO-BP: Gene Ontology Biological Process
HFs: Hair follicles
WGBS: Whole genome bisulfite sequencing
