# Supplementary material for: Whole-genome bisulfite sequencing of goat skins identifies signatures associated with hair cycling
Source: BMC Genomics. 2018 Aug 28;19:638. doi: 10.1186/s12864-018-5002-5 (PMC6114738; doi:10.1186/s12864-018-5002-5)
Supplement: Supplementary file 1 — Figure S1. H&E staining of goat skins at different stage. Figure S2. The correlation between methylation levels and chromosome length, CpG content, the ratio between the observed and expected numbers of CpG sites (CpGo/e), gene number and repeat number. (DOCX 1186 kb) [file 12864_2018_5002_MOESM1_ESM.docx]

**Figure S1. H&E
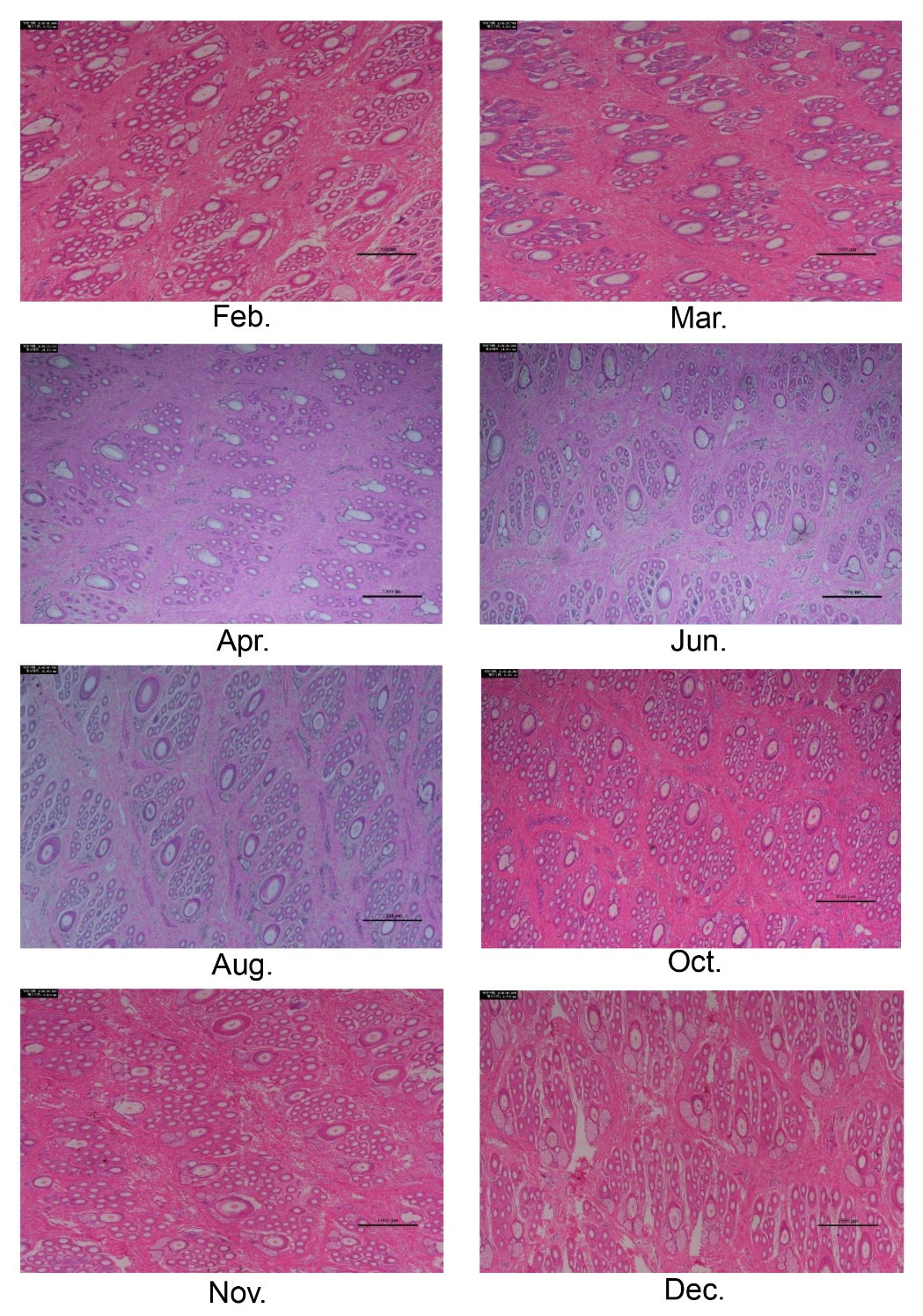
 staining of goat skins at different stage.**

**
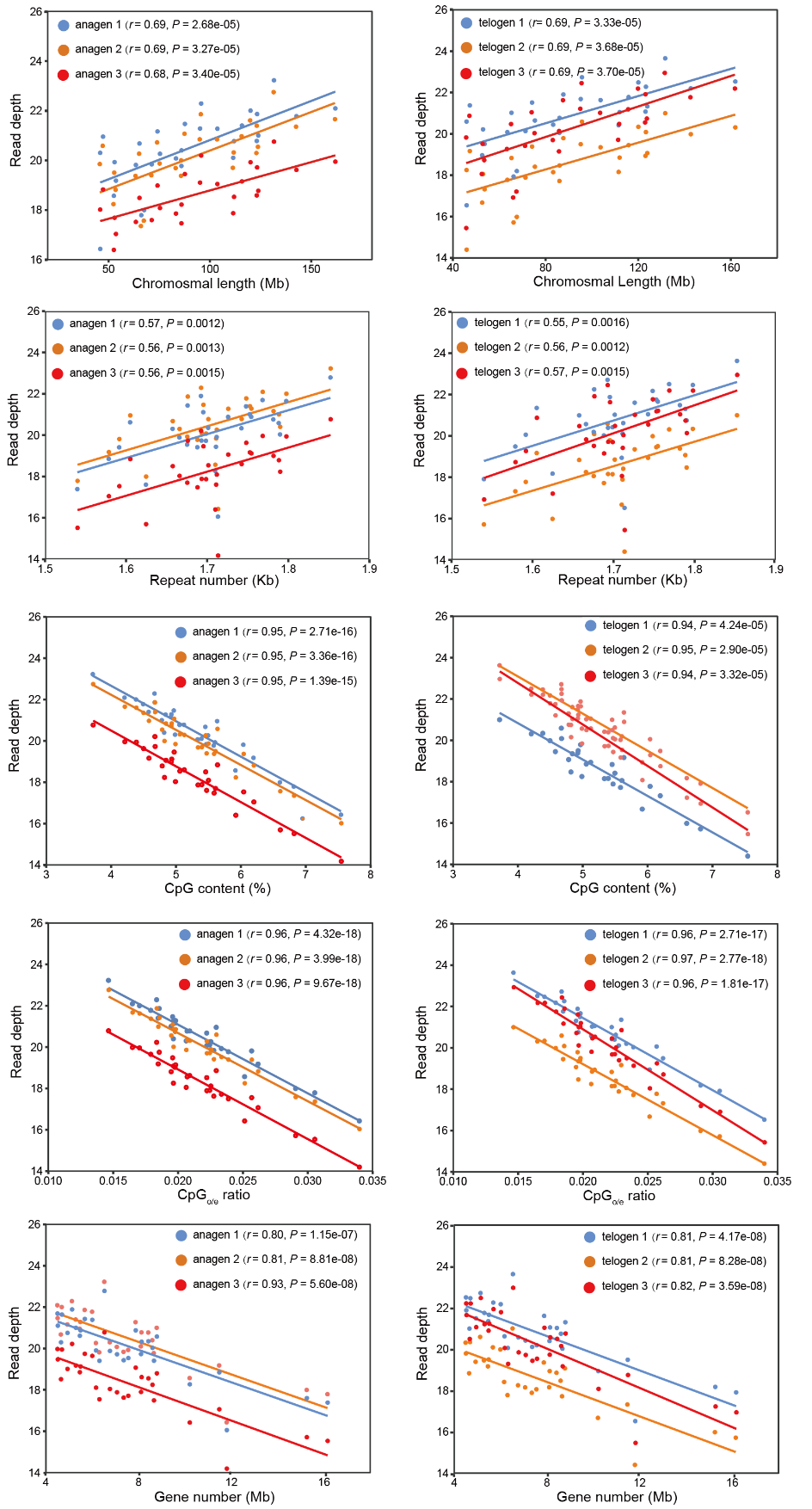
**

**Figure S2.** The correlation between methylation levels and chromosome length, CpG content, the ratio between the observed and expected numbers of CpG sites (CpG_o/e_), gene number and repeat number.

**Table S1** Methylated cytosines in the skins at anagen and telogen stages.

| **Sample** | **Total mC** | **mCG** | **mCHH** | **mCHG** |
| --- | --- | --- | --- | --- |
| anagen_1 | 52,980,374 | 34,733,194 | 13,792,732 | 4,454,448 |
| anagen_2 | 52,565,426 | 34,193,590 | 13,908,280 | 4,463,556 |
| anagen_3 | 45,000,380 | 29,481,714 | 11,715,165 | 3,803,501 |
| telogen_1 | 46,120,521 | 30,444,989 | 11,821,368 | 3,854,164 |
| telogen_2 | 54,653,240 | 35,193,806 | 14,722,724 | 4,736,710 |
| telogen_3 | 43,555,660 | 30,736,724 | 9,748,378 | 3,070,558 |

**Table S2** The proportion of methylated cytosines in goat skins at anagen and telogen stages.

| Samples | mC (%) | mCpG (%) | mCHG (%) | mCHH (%) |
| --- | --- | --- | --- | --- |
| anagen_1 | 4.63% | 59.3% | 1.76% | 1.66% |
| anagen_2 | 4.6% | 58.38% | 1.77% | 1.67% |
| anagen_3 | 3.94% | 50.33% | 1.51% | 1.41% |
| telogen_1 | 4.03% | 51.98% | 1.53% | 1.42% |
| telogen_2 | 4.78% | 60.09% | 1.88% | 1.77% |
| telogen_3 | 3.81% | 52.48% | 1.22% | 1.17% |
